# Widespread gyrovirus co-infections in backyard chickens in Türkiye: molecular and Phylogenetic insights

**DOI:** 10.1007/s11259-026-11241-0

**Published:** 2026-05-02

**Authors:** Mustafa Ozan Atasoy, Turhan Turan, Selda Duran Yelken, Remziye Özbek, Hakan Işıdan

**Affiliations:** 1 Department of Veterinary Virology, Faculty of Veterinary Medicine, Cumhuriyet University, 58140 Sivas, Türkiye; 2 Department of Veterinary Virology, Faculty of Veterinary Medicine, Kastamonu University, 37150 Kastamonu, Türkiye

**Keywords:** Avian gyrovirus 2, Chicken anemia virus, Gyrovirus 3, Gyrovirus 4, Gyrovirus Tu789, Gyrovirus GyV7-SF, Türkiye

## Abstract

**Introduction:**

Gyroviruses are widespread small DNA viruses infecting poultry and other hosts, yet their diversity and coinfection dynamics in backyard systems remained poorly understood.

**Materials and methods:**

Tissue samples from 100 clinically healthy backyard chickens across five provinces in Türkiye, between 2020 and 2021, were screened using species-specific PCR assays targeting six gyroviruses.

**Results:**

The Gyroviral DNA was detected in 90.0% of samples (90/100), with CAV identified in 71.0% (71/100), AGyV2 in 59.0% (59/100), GyV3 in 8.0% (8/100), GyV4 in 22.0% (22/100), GyVTu789 in 19.0% (19/100), and GyV7-SF in 18.0% (18/100) of samples. Co-infections were frequent, most notably between CAV and AGyV2, with a co occurrence rate of 25.6%. Phylogenetic analysis of partial VP1 sequences revealed that circulating strains were largely consistent with global lineages, while also displaying detectable genetic variability, particularly within GyV4. Notably, GyVTu789 and GyV7-SF were identified for the first time in chickens in Türkiye, extending their known geographic range. The high rate of co-detection highlights the concurrent circulation of multiple gyroviruses in backyard poultry populations.

**Conclusions:**

These findings provide a comprehensive overview of gyrovirus diversity and circulation dynamics, pinpointing the need for continued surveillance to better understand their epidemiology and potential impact on poultry health.

**Supplementary Information:**

The online version contains supplementary material available at 10.1007/s11259-026-11241-0.

## Introduction

Gyroviruses are non-enveloped, icosahedral viruses with a single-stranded, negative-sense circular DNA genome of approximately 2.0-2.3 kb, and classified in the family *Anelloviridae* in the current ICTV classification (Rosario et al. 2017; Niu et al. 2019; Loiko et al. 2020; Liu et al. 2022; Kraberger et al. 2025). The genus *Gyrovirus* includes *Gyrovirus chickenanemia* (formerly chicken anemia virus), an important avian pathogen, and thirteen other identified species [*Gyrovirus anas1* (gyrovirus9), *Gyrovirus anas2* (avian gyrovirus 13), *Gyrovirus chauna1* (gyrovirus10), *Gyrovirus fulgla1* (gyrovirus GyV8), *Gyrovirus galga1* (avian gyrovirus 2), *Gyrovirus galga2* (gyrovirus GyV7-SF), *Gyrovirus homsa1* (gyrovirus GyV3), *Gyrovirus homsa2* (gyrovirus Tu789), *Gyrovirus homsa3* (gyrovirus4), *Gyrovirus homsa4* (gyrovirus Tu243), *Gyrovirus hydho1* (ashy storm petrel gyrovirus), *Gyrovirus myferr1* (gyrovirus11), *Gyrovirus phaco1* (pheasant-associated gyrovirus)] (Kraberger et al. 2025). Two additional gyroviruses, *Gyrovirus phasi 1* and *Gyrovirus mega 1*, have recently been reported in wild bird species; however, they remained unclassified so far (Fehér et al. 2022; Wierenga et al., 2023). Gyroviruses have been detected in various hosts such as humans (Phan et al. 2012, 2015), chickens (Chu et al. 2012; Zhang et al. 2013), cats (Niu et al. 2019; Zhang et al. 2014a, b), dogs (Fang et al. 2017), ferrets (Fehér et al. 2014, 2015), mice (Fang et al. 2017), pigeons (Loiko et al. 2020), seabirds (Li et al. 2015; Goldberg et al. 2018; Waits et al. 2018), snakes (Wu et al. 2019), and wild birds (Truchado et al. 2019; Zhang et al. 2023).

Chicken anemia virus (CAV) was isolated from the bursa of chickens in Japan in 1979 for the first time (Yuasa et al. 1979). CAV is prevalent in global chicken populations and has caused significant economic losses in the poultry industry to date. CAV in chickens in Türkiye has been extensively studied and is well documented (Yılmaz et al. 2001; Hadimli et al. 2008; Aşkar and Yıldırım 2011). Avian gyrovirus 2 (AGyV2), the second member of the gyrovirus genus, was first detected in sick chickens in Brazil in 2011 (Rijsewijk et al. 2011). In the same year, another group of researchers in France detected a gyrovirus for the first time in humans (Sauvage et al. 2011), and it was determined that these two viruses had a nucleotide similarity of more than 99%. Gyrovirus GyV3 (GyV3) and gyrovirus 4 (GyV4) were identified in feces for the first time from Chilean and Hong Kong children with acute gastroenteritis in 2012 (Phan et al. 2012). Studies with different animal species have shown that chickens, cats, and ferrets are also infected with GyV3 and GyV4 (Niu et al. 2019; Fehér et al. 2014, 2015; Li et al. 2018; Cibulski et al. 2021). In Türkiye, AvGyV2 has recently been reported at a notably high incidence (73.6%) in diarrheic cats, whereas GyV3 and GyV4 were detected at comparatively lower rates (4.4% and 3.3%, respectively), and no data are currently available from avian species, which are regarded as the primary hosts (Turan et al., 2025). Gyrovirus Tu789 was first identified in the stools of Tunisian children with diarrhea in 2013 (Phan et al. 2013) and was also identified in cats and chickens in the following years (Niu et al. 2019; Cibulski et al. 2021). (Zhang and colleagues 2014) isolated Gyrovirus GyV7-SF from chicken meat sold in markets in the USA (Zhang et al. 2014a, b).

Backyard poultry production is common in the Central and Eastern regions of Türkiye and is often characterised by the close coexistence of chickens with other animal species, including cats, dogs, and large livestock. Such mixed-species environments may facilitate the transmission of infectious agents, particularly in settings where biosecurity measures are inadequate and systematic vaccination programs are lacking. Under these conditions, infections can spread not only among poultry but also between different animal species (Turan et al., 2025). Therefore, continuous surveillance and monitoring of potential etiological agents are essential for the early detection and control of emerging infectious diseases. Therefore, in this study, we investigated the prevalence of gyrovirus species in organ homogenates obtained from backyard chickens in five provinces (Sivas, Malatya, Elazığ, Tunceli, and Bingöl) in the Central and Eastern Anatolian regions of Türkiye. We identified six different gyrovirus species originating from chicken organs, namely CAV, AGyV2, GyV3, GyV4, GyVTu789, and gyrovirus GyV7-SF. In order to better characterise the genetic profiles of the identified gyrovirus strains, partial genome regions were amplified, sequenced, and examined using a variety of molecular techniques. These analyses demonstrated that a number of gyrovirus species are prevalent in backyard chickens, and the strains identified in this investigation exhibited a high degree of genetic diversity.

## Materials and methods

### Sample collection

Organ samples obtained from 100 clinically healthy backyard chickens collected from 5 provinces Sivas, Malatya, Elazığ, Tunceli, Bingöl in the Anatolian regions of Türkiye during 2020–2021 constituted our research material. Internal organ samples were collected from individually managed backyard flocks belonging to different farmers. Each farmer typically kept a single hen with a group of approximately 10–12 chickens, with overall flock sizes ranging from 10 to 20 birds. One adult chicken was sampled from each flock. A total of twenty samples were collected from each province and the trachea, lung, liver, spleen, proventriculus, and intestine were excised from each bird, placed into individual nylon sample bags, and transported to the laboratory on the same day at 4 °C for preparation of 10% tissue homogenates. The tissues were homogenised in sterile PBS at a ratio of saline at a ratio of 1:10 weight to volume using a mechanical homogeniser, and the obtained supernatants were clarified by low-speed centrifugation (3000 x g for 10 min) prior to DNA extraction; clarified supernatants were then stored in 15 mL conical tubes at − 80 °C until further processing.

### DNA extraction and PCR assay detection of gyroviruses

Clarified homogenates were centrifuged at 3,000 × g for 10 min. Total DNA was extracted from the samples using a commercial viral nucleic acid extraction kit (GF-1, Vivantis, Malaysia) according to the manufacturer’s protocol. Novel primer pairs specific for six respective gyrovirus species were designed for this study and are summarised in Table 1, including primer sequences, target genes, annealing temperatures, and expected amplicon sizes. PCR reactions were performed in a final volume of 25 µl. Following an initial denaturation step at 95 °C for 3 min, amplification consisted of 40 cycles at 94 °C for 45 s, annealing at the appropriate temperatures for 45 s, and extension at 72 °C for 1 min, followed by a final extension at 72 °C for 10 min. PCR products were visualised on 1% agarose gels stained with GelRed Nucleic Acid Stain (Merck, USA) under a transilluminator. Samples showing amplicons of the expected size were considered positive.

**Table 1:** Primer sets and PCR conditions for the amplification of six gyroviruses from chicken tissue samples

Primer	Sequence (5’-3’)	Target Gene	Amplicon Size (bp)	Annealing Temp (°C)
CAV 974 F CAV 1328R	GTAGACGAGCTTTTAGGAAG GAGGGCAYGTTATTATCTAG	VP1 gene of Chicken Anemia Virus	355	50
AvGy2-1360 F AvGy2-1969R	CTTGCAGGGGTGCCAATGGT GCTAGGAAATGACCAGGGTGC	VP1 gene of Avian Gyrovirus 2	610	51
Gy3-1101 F Gy3-1744R	ACCCCTATAACGCGATTAACCT TGGTATTGTGGTTTCATTAGCTGG	VP1 gene of Gyrovirus 3	644	51
Gy4-1259 F Gy4-1938R	CTGAAACTTCTGCTTTTAGGGT CGTTTCACTCAATCCAGTAGCT	VP1 gene of Gyrovirus 4	680	50
GyV Tu789-1511 F GyV Tu789-2146R	GCTATAATGGCCGCACAAGA CCAGACGCTTTTTACTCGCAT	VP1 gene of Gyrovirus 6	636	51
GyV7-1007 F GyV7-1559R	AGATGGCAAGACGAGCAAGA TACATCAAGAGCAGTGCCCA	VP1 gene of Gyrovirus 7	553	50

### Integrated genomic, Phylogenetic and statistical analyses

Among the PCR-positive samples, randomly selected amplicons were sequenced, six CAV, five AGyV2, five GyV3, five GyV4, five GyVTu789, and six GyV7-SF, all corresponding to partial VP1 gene regions. PCR products were purified through an external service provider prior to sequencing, and bidirectional Sanger sequencing was performed using the same primer pairs.

Sequence quality was initially assessed by examining chromatograms to confirm base-calling accuracy and resolve ambiguous peaks. Subsequently, multiple sequence alignments for each respective virus were performed using the AliView alignment program. Phylogenetic analyses were conducted in MEGA X using the maximum likelihood (ML) method (Kumar et al. 2018). The Bayesian Information Criterion (BIC) was used to determine the most appropriate nucleotide substitution model. Phylogenetic trees were then constructed based on the selected models for each dataset as follows: the Kimura 2-parameter model with gamma distribution (+ G) for CAV and AGyV2, as well as for GyV3; the Jukes–Cantor model for GyV7-SF; the Tamura 3-parameter model with invariant sites (+ I) for GyV4; and the Tamura–Nei model with gamma distribution (+ G) for GyVTu789, with 1,000 bootstrap replicates. Nucleotide identity (nt) values were calculated using the SIAS online tool (http://imed.med.ucm.es/Tools/sias.html).

A heatmap and a stacked bar chart illustrating positivity rates were generated in GraphPad Prism (GraphPad Software, San Diego, CA, USA). The UpSet plot illustrating the co-occurrence patterns of detected viruses was generated using RStudio v.2024.09.0 + 375 (Posit Software PBC, MA, USA), based on sample IDs assigned to each virus-positive group.

## Results

### Prevalence of gyroviruses in chicken tissue homogenate samples

**Fig. 1:**
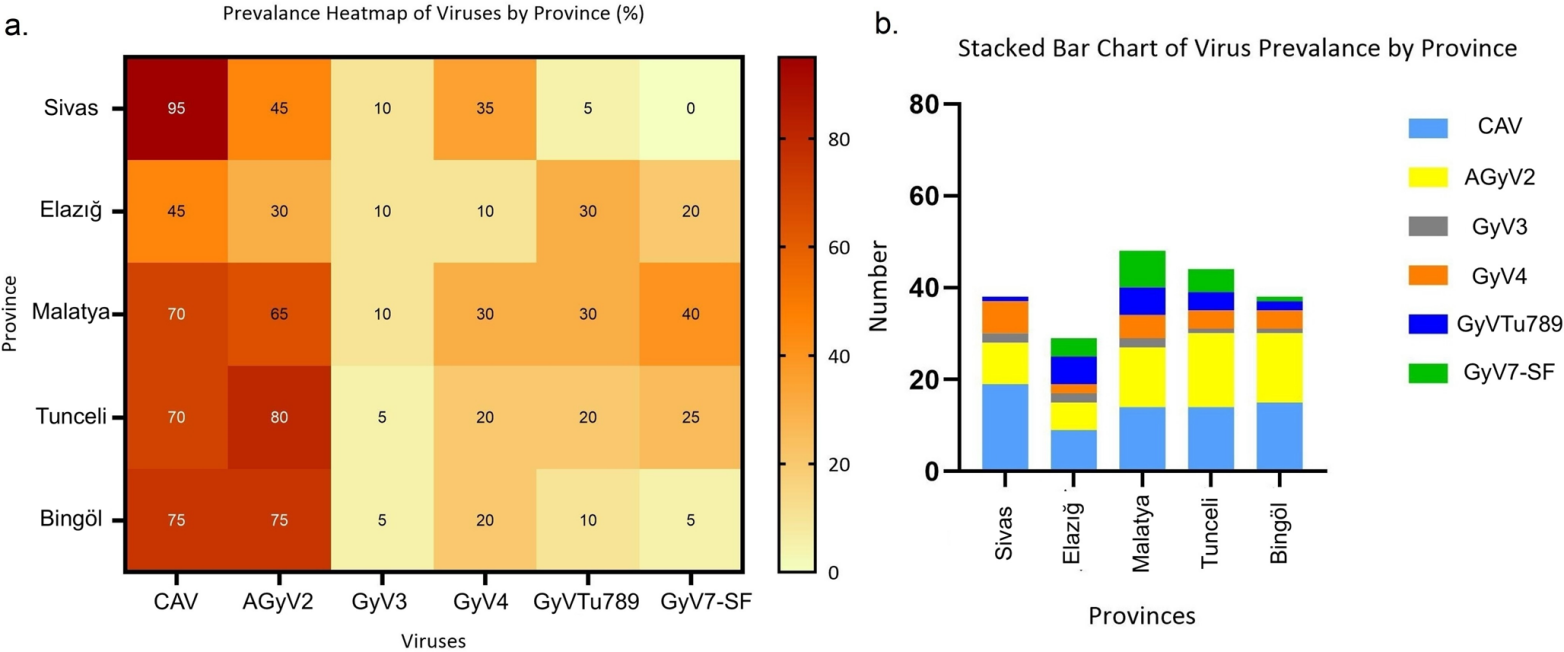
Heatmap and stacked bar chart illustrating the prevalence and distribution of six gyroviruses in backyard chickens across five provinces (Sivas, Elazığ, Malatya, Tunceli, and Bingöl). (a) The heatmap represents percentage positivity rates for each virus by province and (b) stacked bar chart shows the numerical distribution of virus-positive samples, highlighting differences in single and multiple infections among provinces

In the present study, at least one type of chicken gyrovirus was detected in 90 out of 100 organ tissue homogenate samples. Among these, CAV was identified in 71.0% of the samples, AGyV2 in 59.0%, GyV3 in 8.0%, GyV4 in 22.0%, GyVTu789 in 19.0%, and GyV7-SF in 18.0%. The distribution of positivity rates according to the provinces from which the tissue homogenate samples were obtained is presented in Fig. 1.

**Fig. 2:**
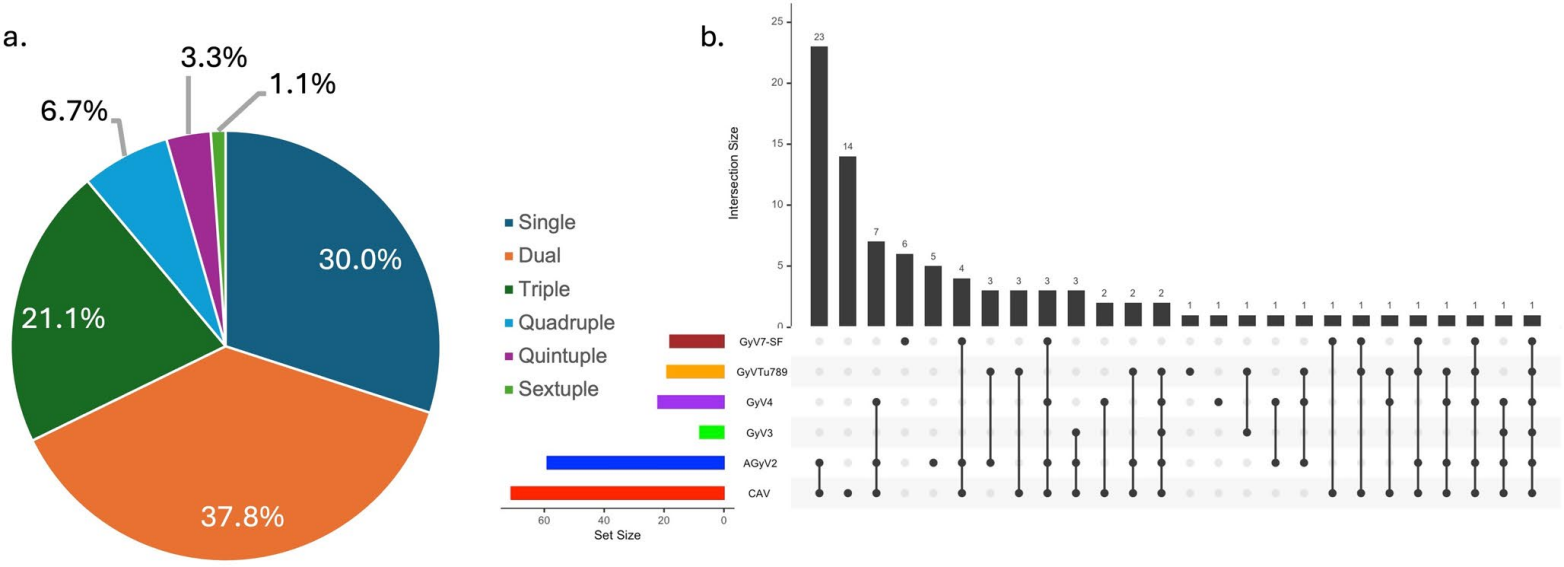
**(a)** Pie chart illustrating the proportion of single and multiple gyrovirus infections detected in backyard chickens, including single, dual, triple, quadruple, quintuple, and sextuple infection patterns. **(b)** UpSet plot depicting the intersection sizes and co-occurrence patterns among five detected gyroviruses (CAV, AGyV2, GyV4, GyVTu789, and GyV7), highlighting the most frequent virus combinations observed in the sampled population

Out of the 100 chickens examined, 27 had infections caused by a single gyrovirus. The most common was CAV, found in 14 chickens, followed by GyV7-SF in six, AGyV2 in five, and both GyV4 and GyV7-SF in one chicken each. Dual infections were even more frequent, affecting 34 (37.8%) chickens, the most common combination was CAV and AGyV2, detected together in 23 (25.6%) chickens. Other combinations included pairs like CAV with GyVTu789, AGyV2 with GyVTu789, and GyV3 with GyVTu789. Triple infections were detected in 19 chickens, with the most frequent combination being CAV, AGyV2, and GyV4. Some chickens also had infections involving four or even five different gyroviruses. Our findings also reveal the existence of a rare case where all six viruses (CAV, AGyV2, GyV3, GyV4, GyVTu789, GyV7-SF) were detected simultaneously (*n* = 1; 1.1%) (see Fig. 2a and b).

### Molecular analysis of chicken anemia virus

Nucleotide and amino acid (aa) similarities ranged from 90.9% to 100%, and 94.1% to 100.0%, among sequences of the CAV strains in the phylogenetic tree, and 96.0% to 100%, and 96.6% to 100.0% among each other, respectively, which indicating high genetic similarity. Strain ChAV/TUR/94 (MN717260) sequence was identical to reference strains detected in cat samples from Türkiye PQ281758/-77/-60, and chicken samples from South Korea (MW091334 – MW091337). Other sequences showed 100% aa similarity to reference strains detected from pigeon (PQ281778, PQ281779) and cat (PQ281759, PQ281761, PQ281762, PQ281775) stool samples from Türkiye.

**Fig. 3:**
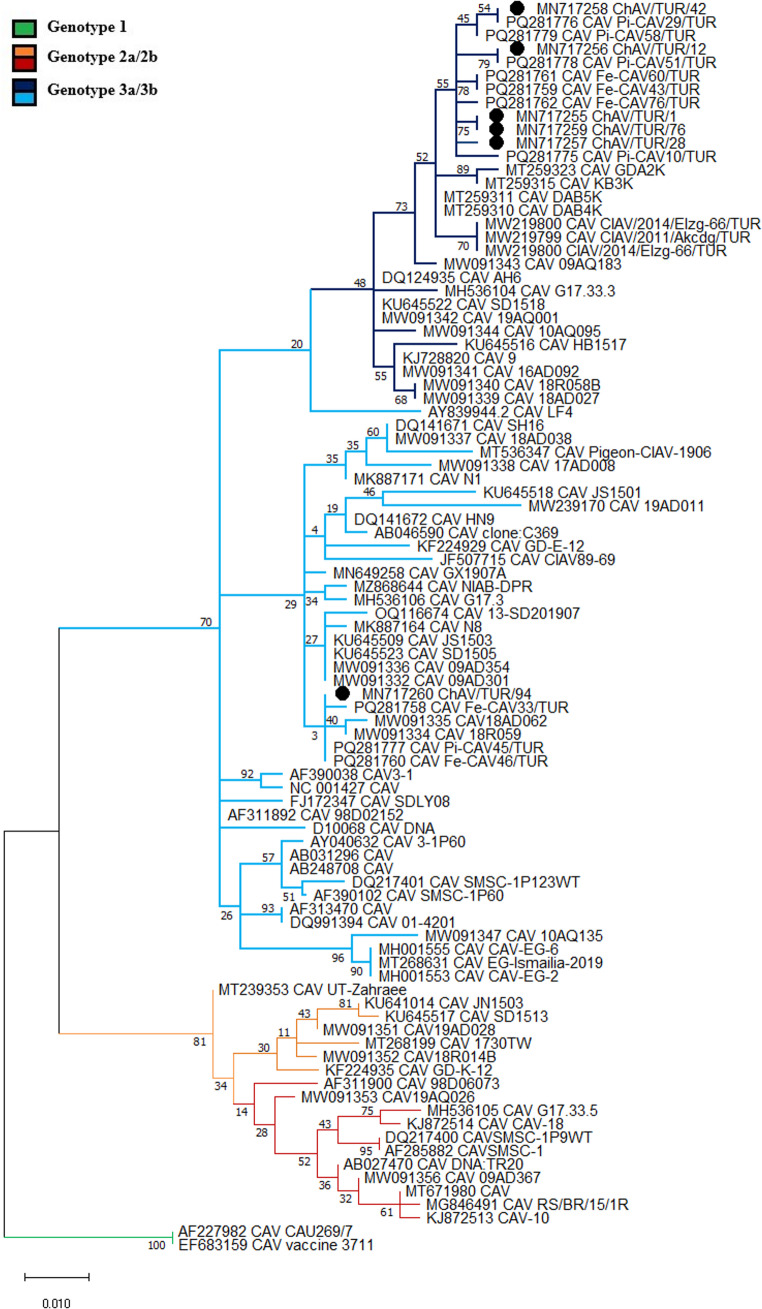
Phylogenetic relationships of Chicken Anemia Virus (CAV) based on the VP1 gene sequences. Isolates clustered into the three major genotypes: Genotype 1 (green branches), Genotype 2a/2b (orange-red branches), and Genotype 3a/3b (blue-navyblue branches). Black-filled circles indicate the CAV field strains obtained in this study. The tree demonstrates the evolutionary relationships between Turkish CAV isolates and global reference sequences

**Fig. 4:**
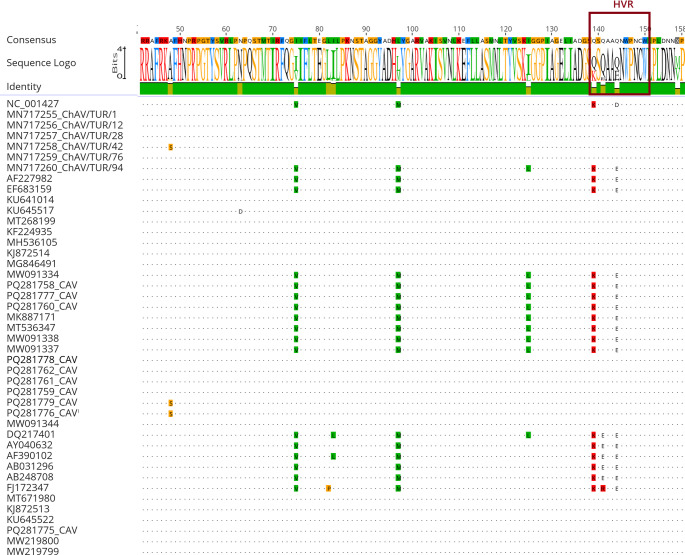
Multiple sequence alignment based on the deduced amino acid sequences of the VP1 gene. The alignment includes all Turkish strains identified in this study together with selected reference strains retrieved from GenBank. The hypervariable region (HVR) is indicated by a boxed area

Phylogenetic analysis revealed that CAV isolates were classified into three major genotypes: Genotype 1, Genotype 2a/2b, and Genotype 3a/3b. Pairwise comparison with reference strains showed that among CAV sequences five strains, ChAV/TUR/1, ChAV/TUR/12, ChAV/TUR/28, ChAV/TUR/42 and ChAV/TUR/76 (MN717255–MN717259, respectively) clustered with the Genotype 3a, while ChAV/TUR/94 (MN717260), a strain from Bingöl province, were with in the Genotype 3b (Fig. 3). When compared to the reference sequence, strain ChAV/TUR/1 (MN717255), ChAV/TUR/12 (MN717256), ChAV/TUR/28 (MN717257), and ChAV/TUR/76 (MN717259) exhibited the amino acid variations compared to the reference sequence: V75I, M97L, K139Q, D144Q, V157M. Additionally, in ChAV/TUR/42 (MN717258), an Ala-to-Ser substitution was detected at position 48. This substitution, apart from the common changes described above, was not identified in the other strains analysed in this study and was observed only in a limited number of reference strains (Fig. 4). The ChAV/TUR/94 (MN717260) strain exhibited amino acid substitutions distinct from those identified in the other sequences analysed in this study. Specifically, the substitutions I125L, D144E, and V157M were detected, while no variations were observed at positions 75, 97, or 139. The substitutions at positions 144 and 157 were also present in several other strains, whereas the I125L substitution demonstrated a more restricted distribution (Fig. 4).

### Molecular analyses of other gyroviruses

Among the positive gyrovirus samples, five AGyV2, five GyV3, five GyV4, five GyVTu789, and six GyV7-SF samples were successfully sequenced. The partial VP1 sequences generated in this study were deposited in GenBank under the accession numbers PZ056908–PZ056912 (AGyV2), PZ056913–PZ056917 (GyV3), PZ056918–PZ056922 (GyV4), PZ056923–PZ056927 (GyVTu789), and PZ056928–PZ056933 (GyV7-SF).

#### AGyV2 (*Gyrovirus galga1*)

Nucleotide sequence analysis of the chicken-derived AGyV2 isolates revealed 92.8–99.8% identity among the study sequences. Amino acid sequence comparisons demonstrated a higher degree of conservation, ranging from 98.0% to 100%. When compared with GenBank reference sequences, AvGyV2/TUR/1, AvGyV2/TUR/35, and AvGyV2/TUR/48 exhibited the highest similarity to previously reported Turkish strains (PQ281780 and PQ281782), showing 99.7–100% nucleotide identity and 100% amino acid identity^1^. AvGyV2/TUR/15 showed complete nucleotide and amino acid identity with the Turkish strain PQ281763, whereas AvGyV2/TUR/75 displayed 100% identity at both nucleotide and amino acid levels with PQ281766 and PQ281783.

All sequences encoded a 203–amino acid fragment of VP1, and no insertions or deletions were detected. Relative to the reference strain NC_015396, three isolates (AvGyV2/TUR/1, AvGyV2/TUR/35, and AvGyV2/TUR/48) differed by only two amino acid substitutions (R212K and G242R, according to full-length VP1 numbering), while AvGyV2/TUR/75 and AvGyV2/TUR/15 contained additional substitutions. The R212K and G242R substitutions were the most frequently observed changes and are widely present among GenBank AGyV2 sequences. Other substitutions, including S154A, A270S, V288Q, G293Q, and Q310E, were detected at varying frequencies in reference sequences, whereas the V288I substitution identified in AvGyV2/TUR/15 was rare.

**Fig. 5:**
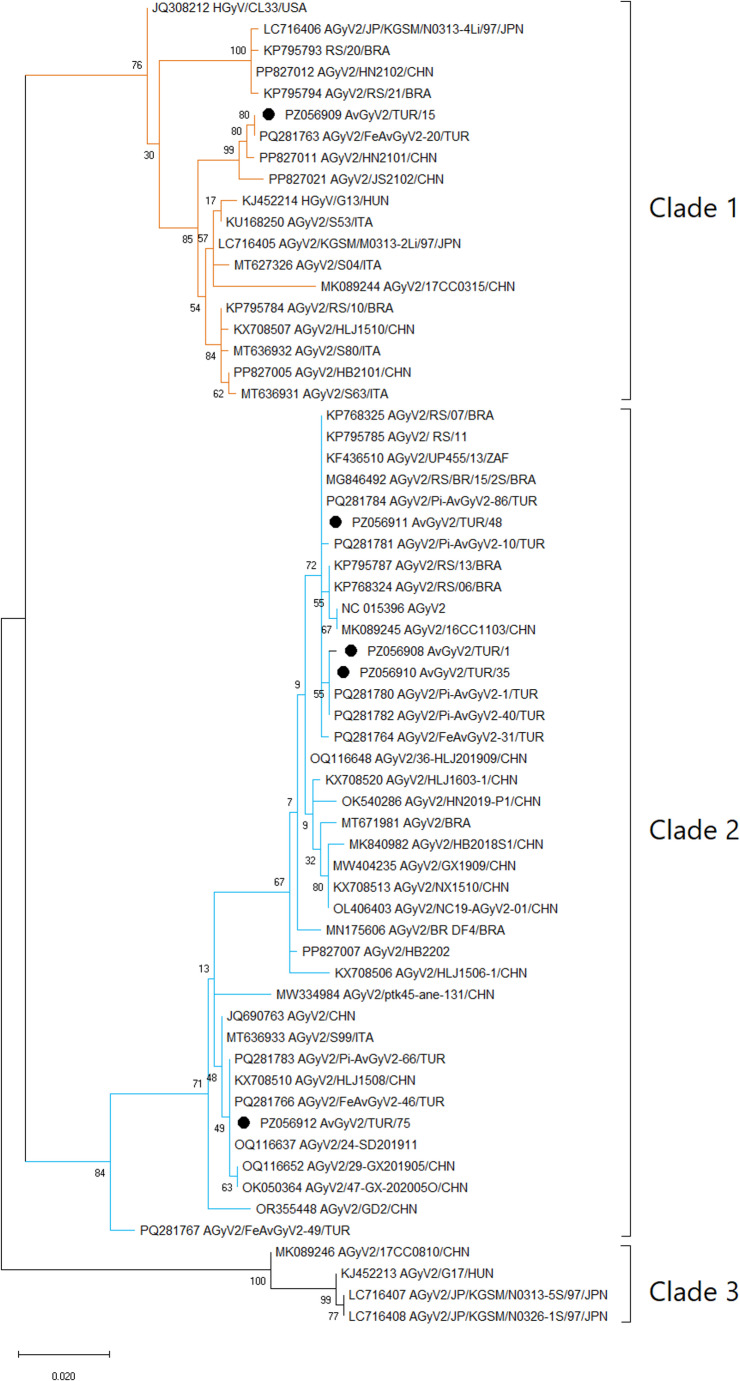
Maximum-likelihood phylogenetic tree of avian gyrovirus 2 (AGyV2) constructed based on partial VP1 sequences. The tree reveals three major clades (Clade 1–3), in agreement with previously reported classifications. Sequences generated in this study are indicated by black circles

Phylogenetic analysis revealed that AGyV2 sequences were distributed into three major clades (Clade 1–3). Most isolates obtained in this study clustered within Clade 2, forming closely related subclusters with short branch lengths and moderate to high bootstrap support. In contrast, AvGyV2/TUR/15 was positioned within Clade 1, indicating genetic heterogeneity among the detected strains (Fig. 5).

#### Analysis of GyV3 (*Gyrovirus homsa1*)

**Fig. 6:**
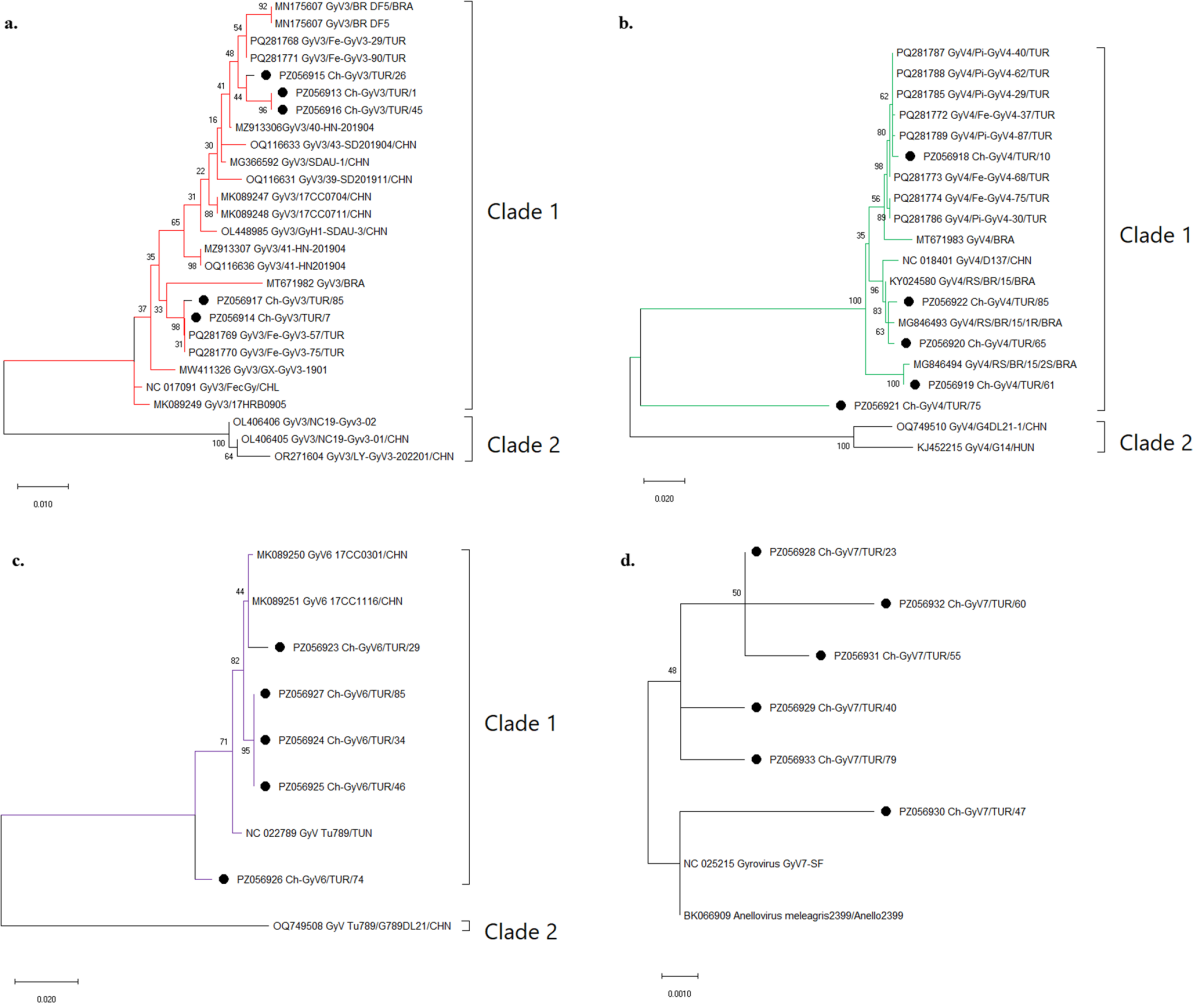
Maximum likelihood phylogenies of Gyrovirus 3, 4, GyVTu789 and GyV7 (a–d). Phylogenetic trees were reconstructed using the maximum likelihood method, with the best-fit nucleotide substitution models selected by Find Best Model (K2P + G). Each panel represents a distinct Gyrovirus type: **(a)** Gyrovirus 3, **(b)** Gyrovirus 4, **(c)** GyVTu789, and **(d)** GyV7. Bootstrap values (≥ 70%) are shown at major nodes

The five chicken-derived GyV3 isolates exhibited high nucleotide identity among themselves, ranging from 98.3% to 100%, while amino acid identities ranged from 99.1% to 100%. Comparison with GenBank reference sequences showed that these isolates were most closely related to previously reported poultry-origin strains, with 99.2–100% nucleotide identity and 99.5–100% amino acid identity. Lower similarity values were observed when compared with non-poultry-derived reference sequences. Amino acid sequence alignment of a 214–amino acid region identified nine variable positions among the aligned sequences, indicating a high level of conservation. Phylogenetic analysis demonstrated that all study isolates clustered within Clade 1 together with poultry-origin Brazilian references and cat-origin Turkish reference sequences. The isolates formed a compact cluster supported by moderate to high bootstrap values (Fig. 6a).

#### Analysis of GyV4 (*Gyrovirus homsa3*)

In contrast to the other gyroviruses, GyV4 isolates displayed greater genetic variability. Pairwise nucleotide identities among the chicken-derived sequences ranged from 85.4% to 99.3%, whereas amino acid identities ranged from 94.2% to 99.6%. The highest similarities were observed with poultry-origin reference strains (87.6–99.7% nucleotide and 95.0–99.7% amino acid identity), while lower similarity values were detected in sequences derived from other hosts. Alignment of a 226–amino acid region revealed 24 variable sites, reflecting a comparatively higher level of genetic diversity. No insertions or deletions were detected. Phylogenetically, most chicken-derived isolates were positioned within Clade 1 and clustered with poultry-associated Chinese reference sequences, demonstrating close genetic relatedness based on branch lengths and clustering patterns (Fig. 6b).

#### Analysis of GyVTu789 (*Gyrovirus homsa2*)

The GyV (GyVTu789) isolates were highly conserved, exhibiting nucleotide identities of 97.6–100% and amino acid identities of 98.4–100% among themselves. Comparison with GenBank reference sequences revealed the highest similarity with previously reported GyVTu789 strains, showing 98.1–99.5% nucleotide identity and 98.8–100% amino acid identity, while slightly lower similarities were observed with more distantly related sequences. Furthermore, amino acid alignment across a 212–amino acid region identified eight variable positions, confirming limited variability and the absence of insertions or deletions. Phylogenetic analysis showed that most isolates grouped within Clade 1 together with Turkish, Brazilian, and some Chinese reference strains (see Fig. 6c).

#### Analysis of GyV7-SF (*Gyrovirus galga2*)

Sequence analysis of the GyV7-SF region demonstrated high conservation among the chicken-derived isolates, with nucleotide identities ranging from 98.7% to 99.8% and amino acid identities between 99.0% and 100%. All isolates showed consistently high similarity to previously reported GyV7-SF reference strains, with 99.3–99.6% nucleotide identity and 99.2–100% amino acid identity. Alignment of a 184–amino acid region identified only seven variable sites, representing the lowest variability among the analyzed gyroviruses. No insertions or deletions were detected. In the phylogenetic tree, all chicken-derived isolates clustered tightly with the GyV7-SF reference sequence, forming a compact group characterized by short branch lengths and strong bootstrap support, reflecting high genetic conservation within this genomic region (Fig. 6d).

## Discussion

In this study, samples taken from chicken organ tissues in the Central Anatolia region of Türkiye showed the co-existence of six distinct gyrovirus types (CAV, AvGyV2, GyV3, GyV4, GyVTu789, and GyV7-SF). The detection of at least one gyrovirus in the majority of samples (90.0%) indicates that these viruses also widely existed in non-commercial flocks. This result is consistent with studies conducted in recent years on anellovirus studies worldwide (Zhang et al. 2013; Rosario et al. 2017; Niu et al. 2019; Loiko et al. 2020; Cibulski et al. 2021). Additionally, it is noteworthy that a strong co-prevalence was found, specifically between AGyV2 and CAV. CAV infection can cause severe immunosuppression, anemia, and bone marrow failure in chicks (Fatoba and Adeleke 2019), whereas in adult chickens, it is typically subclinical but still contributes to immunosuppression, thereby facilitating the establishment of concurrent infections, such as infectious bronchitis (IB) (Toro et al. 2006). More importantly, subclinical CAV infection may contribute to vaccine failure against high-mortality diseases such as Newcastle disease, leading to increased susceptibility of intensively managed flocks to severe infections (Wu et al. 2026). In our study, CAV was detected at a notably high rate (71.0%) in Central and Eastern Anatolian regions. Aşkar and Yıldırım (2011) reported no detectable active Chicken Anemia Virus infection by PCR in healthy commercial broiler flocks in Central Anatolia; nevertheless, all unvaccinated breeder flocks were seropositive, suggesting prior exposure to the virus (Aşkar and Yıldırım 2011). Similarly, Hadimli et al. (2008) conducted a more comprehensive analysis of CAV presence in infected commercial broilers across various provinces in Türkiye, revealing a high prevalence of the virus and its frequent coexistence with other viral diseases, including IB and Marek’s disease (Hadimli et al. 2008). Altogether, the pathogenetic characteristics of CAV, particularly its tropism for hematopoietic tissues such as the bone marrow and its capacity to induce persistent, often subclinical infections, may account for the high detection rates observed in poultry populations. This persistent immunosuppressive state likely facilitates viral co-infections and contributes to the frequent co-occurrence of CAV with other pathogens.

The highest rate of viral co-infection was observed between CAV and AGyV2. AGyV2 has been reported to exhibit high prevalence and notable genetic diversity, with co-infections considered epidemiologically significant, and it may also co-evolve through recombination events (Phan et al. 2012; Liu et al. 2022). To date, no experimental study has specifically investigated co-infection between CAV and AGyV2; however, evidence from studies involving CAV and other gyroviruses suggests potential synergistic effects (Yang et al. 2023). Additionally, in cats, severe infections such as feline panleukopenia virus have been associated with increased detection of AGyV2 (Turan et al. 2025). Accordingly, the frequent co-detection of CAV and AGyV2 observed in the present study may reflect interactions that enhance viral persistence and transmission, particularly under conditions of CAV-induced immunosuppression.

Phylogenetic analysis demonstrated that the CAV sequences clustered within the Genotype 3a/3b group, consistent with previous reports from Türkiye and other regions where this genotype has predominated in recent years (Sun et al. 2023; Song et al. 2024). Variations in pathogenicity have been suggested to be associated with point mutations in the VP1 protein. The proximity of some isolates to the Genotype 1 lineage, supported by high bootstrap values, may indicate possible reintroductions of the virus, commercial animal movements, or transmission from different geographical sources. This is supported by the clustering of highly similar strains from different provinces, as well as the presence of a distinct IIIa strain from a separate province, particularly exhibiting variation in the HVR region which previously described elsewhere (Renshaw et al. 1996; Todd DScott et al. 2002) Taken together, our findings highlight the importance of evaluating vaccination strategies and flock management practices together with recent phylogenetic data.

AGyV2, initially identified in chickens and later reported as human gyrovirus, is a small circular single-stranded DNA virus belonging to the genus *Gyrovirus*, the prototype genome represented by clone Ave 3 (NC_015396) (Phan et al., 2012; Biagini et al., 2013 ). Since its first description, AGyV2 has been detected in poultry flocks from different geographic regions, indicating its widespread distribution and sustained circulation in chicken populations (Rijsewijk et al. 2011; Chu et al. 2012). In addition, AGyV2 DNA has been identified in poultry-derived products and in various human sample types, raising interest regarding potential exposure routes, although no clear association with disease has been established (Phan et al. 2012). To date, however, molecular data on AGyV2 from poultry in Türkiye remained limited. In this context, amino acid–level sequence analyses are widely used to place newly detected AGyV2 strains within the global diversity framework and to assess whether observed substitutions represent dominant circulating variants or rare lineage-specific changes (Rijsewijk et al. 2011; Chu et al. 2012).

In this study, molecular analysis showed that several chicken-derived AGyV2 sequences were highly conserved. All sequences encoded a protein of the same length (203 amino acids). Three isolates (AvGyV2/TUR/1, AvGyV2/TUR/35, and AvGyV2/TUR/48) differed from the reference strain by only two amino acid substitutions (R212K and G242R), both of which were very common among other available sequences, suggesting that these changes represent widely circulating variants rather than study-specific mutations. Two isolates (AvGyV2/TUR/15 and AvGyV2/TUR/75) showed additional amino acid substitutions; however, most of these changes were also frequently observed in previously reported sequences and therefore fall within the known genetic diversity of AGyV2. The V288I substitution detected in AvGyV2/TUR/15 was rare among GenBank sequences and may indicate limited local variation, although its biological significance is currently unknown. Overall, no novel amino acid substitutions were identified, and the chicken-derived isolates showed high similarity to globally circulating AGyV2 strains, indicating limited protein-level variation. Amino acid positions are reported according to the full-length VP1 numbering of the reference strain NC_015396.

GyV3 was first identified in human fecal samples and was later identified in poultry-related materials, suggesting an association with avian origin, while GyV4 was subsequently detected in human samples in Europe, further expanding the known diversity of gyroviruses (Phan et al. 2012; Chu et al. 2012). The pathogenic role of GyV3 and GyV4 remained unclear; however, their repeated detection in poultry products and human samples has increased interest in their epidemiology and genetic characteristics. Therefore, amino acid–level sequence analysis is useful to compare locally detected GyV3 and GyV4 strains with globally reported sequences. Our study shows that the lower detection rates of GyV3 and GyV4 indicate that the circulation of these viruses within the flock is more limited. However, the fact that these two viruses have been isolated not only from chickens but also from cats, ferrets, and other species strengthens the possibility of environmental transmission (Fehér et al. 2014; Niu et al. 2019; Li et al. 2018). Similarly, simultaneous detection of multiple gyrovirus species co-occurring with anelloviruses has been reported in cats with diarrhea (Turan et al. 2025).

One of the most noteworthy findings of this study was the detection of GyVTu789 and GyV7-SF. GyVTu789 strain Tu789 was first identified in fecal samples from Tunisian children (Phan et al. 2013) and subsequently reported in cat samples and chicken products (Niu et al. 2019; Cibulski et al. 2021). Since the first identification of GyV7-SF in chicken meat purchased from retail markets in the United States, the virus has also been detected in turkey samples in a recent metagenomic study (Zhang et al. 2014a, b; Buck et al. 2024). To the best of our knowledge, this study represents the first report of both GyVTu789 and GyV7-SF in Türkiye, thereby expanding the known geographic distribution of these gyroviruses. The detection of these viruses in organ tissues in this study suggests that they may have been circulating undetected for a long time or may have been introduced into the region through the environmental factors. The detection of multiple gyroviruses in a single sample indicates that these viruses are capable of co-occurring within the same environment or host.

Molecular and phylogenetic analyses showed that the isolates from Türkiye are similar to reference strains reported in other countries. This suggests that gyroviruses may spread between regions through global trade, biological products, or migratory birds. The diversity seen in the VP1 gene agrees with previous studies, and this gene region is known to be important for antigenic properties, host adaptation, and virus evolution (Fatoba and Adeleke 2019; Niu et al. 2019).

There were several shortcomings in this study. First, the main objective was virus detection and co-occurrence rather than region-specific prevalence, which is difficult to estimate in backyard systems where diseased birds are often culled before sampling; therefore, overall prevalence could not be determined. Second, the study focused on virus detection and genomic insights, and primer sensitivity and specificity were not evaluated, as methodological performance and comparison were beyond the scope. However, this remains a limitation and should be addressed in future studies, particularly through the development and application of quantitative approaches such as real-time PCR or LAMP assays. Such approaches would enable more comprehensive data and provide a clearer understanding of viral loads, especially in asymptomatic animals that may act as carriers of multiple viruses.

## Conclusion

This study demonstrated that backyard chickens in Central and Eastern Türkiye harbored multiple and genetically diverse gyrovirus species. The detection of different gyroviruses within organ homogenates indicated that these viruses could coexist and circulate simultaneously in the same host population and merits further investigation. Furthermore, the detection of gyroviruses previously reported in humans emphasises the need for continued monitoring to understand their spread and diversity in backyard poultry.

## Supplementary Information

Below is the link to the electronic supplementary material.

Supplementary Material 1

## Data Availability

The partial VP1 sequences obtained in this study were deposited in the GenBank database under the accession numbers: PZ056908–PZ056933. Detailed information on the sequences, including strain identifiers and sampling locations, is provided in Supplementary Table S1.

## Footnotes

1 Strain identifiers (e.g., AvGyV2/TUR/1) are retained as originally deposited in NCBI and are not retroactively updated to reflect the standardised abbreviation AGyV2.
